# Effects of aqueous extract of celery (*Apium graveolens *L.) leaves on spermatogenesis in healthy male rats

**Published:** 2015

**Authors:** Ameneh Hardani, Mohammad Reza Afzalzadeh, Ashraf Amirzargar, Esrafil Mansouri, Zakieh Meamar

**Affiliations:** 1 *Department of Public Health, Student at the Faculty of Hygiene Sciences, Ahvaz Jundishapur University of Medical Sciences, Ahvaz, Iran *; 2 *Department of Physiology, Student at the Faculty of Veterinary Medicine, Shahid Chamran University, Ahvaz, Iran*; 3 *Department of Physiology and Diabetes Research Center, Faculty of Medical Sciences, Ahvaz Jundishapur University of Medical Sciences, Ahvaz, Iran*; 4 *Department of Histology Research Center, Faculty of Medical Sciences, Ahvaz Jundishapur University of Medical Sciences, Ahvaz, Iran*; 5 *Department of Engineering Environmental Health, Faculty of Hygiene Sciences, Ahvaz Jundishapur University of Medical Sciences, Ahvaz, Iran*

**Keywords:** *Celery*, *Leaves*, *Testis*, *Spermatozoid*, *Spermatogenesis*, *Rat*

## Abstract

**Objectives:**

Nowadays, a lot of attention has been paid to the therapeutic properties of herbs, including evaluation of the effects of these plants on fertility in laboratory animals. *Apium graveolens* L. (celery) has been widely used in traditional medicine for treatment of various disorders including impotency. Therefore, this study was designed to investigate the effects of aqueous extract of *A. graveolens* on testicular tissue and spermatogenesis in healthy male rats.

**Materials and Methods:**

In this research, 24 apparently healthy male rats were divided into three groups, including eight rats in each. The first group as control received only distilled water 1 ml/animal/day. The second and third groups orally received 100 and 200 mg/kg b.w. of the extract, respectively, for 30 days. The day after the last administration of the extract, the rats were sacrificed, the testes were removed entirely, and the morphometric studies were carried out. Epididymal sperm count and histological studies of testicular tissue were conducted.

**Results:**

The comparison between the treated and control groups revealed a remarkable increase in the seminiferous tubules diameter, testes volume (p≤0.001), and the number of spermatogonia, primary spermatocytes and spermatozoa. Furthermore, the increase in the number of spermatids and epididymal weight were only significant at high doses of the extract (p≤ 0.05).

**Conclusions:**

The results from this study indicated that administration of celery leaf extract may improve spermatogenesis process and also be useful for some sperm fertility parameters.

## Introduction

In many developed countries, herbal and traditional medicines are applied in treating many diseases, including experimental wound healing, hypertension, diabetes, and reproductive performance (Modaresi, 2012 ▶). In recent years, much attention has been paid to studying the effects of different plants on the fertility of laboratory animals. These studies have provided valuable information (Tasdighi et al., 2012 ▶).

Celery (*Apium graveolens* L.) is a plant, belonging to the parsley descent (Umblliferace), an herbaceous, biennial, and branched stem plant, with a height of 20 to 60 cm (Nasri et al., 2009 ▶). Celery leaves contain different compounds such as valerophenone (19.90%), 1-dodecanol (16.55%), 9-octadecanoic acid, and methyl ester (4.93%) (Nagella et al., 2012 ▶). The juice of celery leaves and roots possesses effective biochemical parameters, such as reduced glutathione content, catalase, xanthine oxidase, glutathione peroxidase, and peroxidase activities which affect the intensity of lipid peroxidation in homogenized liver and blood. When this extract is used in combination with doxorubicin, a protective effect is induced against it (Kolarovic et al., 2009 ▶). Celery extract causes a significant reduction in serum levels of total cholesterol and low density lipoprotein (LDL), in individuals. It also increases the hepatic triglyceride by reducing the activity of hepatic triacylglycerol lipase (Mansi et al., 2009 ▶). Moreover, fatty acids are essential for proper function of spermatogenesis (Taati et al., 2011 ▶). These extracts have several effects such as anti-inflammatory, anticancer, anti-hepatotoxic, anti-hypercholesterolemic, analgesic, anti-bacterial, and anti-spasmodic (Modaresi et al., 2012 ▶) as well as effects on smooth muscle contractions (Gharib Naseri et al., 2007 ▶). It has been known as an appetite and libido stimulant in traditional medicine (Khosravi, 2006 ▶) and it increases the secretion of breast milk, as well (Fluke, 2005 ▶). Celery extracts have protective effects against sodium valproate in testes (Hamza and Amin, 2007 ▶). Moreover, it has a protective role in the testes and amplifies the sperm parameters (Kerishchi et al., 2011 ▶). Therefore, the present study aimed to investigate the possible effects of celery extract on spermatogenesis and histological changes of testes in male rats.

## Materials and Methods

**Preparation of the aqueous extract**

To prepare the celery extract, the leaves were purchased from one of the vegetable stores in Ahvaz, Iran. A voucher specimen was deposited at the herbarium of Shahid Chamran University of Ahvaz. The leaves were then dried in the shade and milled, and powders were stored in a refrigerator (4 C) until extraction. Fifty g of celery leaf powder was mixed in 200 ml of distilled water in a Soxhlet apparatus for 24 hr. Then, the mixture was filtered with a filter paper (Whatman No.1) and the filtrate was concentrated in a rotary evaporator. At the end, the concentrated extract was stored at 4 °C until use. The extract was diluted with distilled water and administered according to the required concentration.

**Experimental animals**

This study was conducted experimentally in Physiology Research Center, Ahvaz Jundishapur University of Medical Sciences (AJUMS). Twenty four adult male *Wistar* rats (8 weeks old), weighing (170-220 g) were obtained from the animal reproduction andbreeding center of AJUMS. Procedures involving animals and their care were conducted in conformity with Committee of Ethics in Research of the AJUMS. The animals were kept in standard polycarbonate cages, carpeted with wood chips, in an appropriate environmental condition, and temperatures around 22±2 °C, periods of 12 h light/12 h dark cycle, with free access to water and standard food (*ad libitum*) were considered, as well. All groups of the experiment were maintained in their cages for 24 hr to get accustomed to the environment. These conditions were maintained throughout the experiment. The cages were disinfected twice a week and the wood chips were replaced every two days.

**Experimental protocol**

The animals were randomly divided into three groups (eight rats each) and classified as follows:

Group 1: control, received 1 ml/animal/day distilled water (Mokhtari and Zanboori, 2011 ▶).

Group 2: received 100 mg/kg/day of celery leaf extract (Ramezani et al., 2009 ▶).

Group 3: received 200 mg/kg/day of celery leaf extract (Hamza and Amin, 2007 ▶).

Forty-eight days are required to complete the whole spermatogenic step in rats (Krinke, 2000 ▶), but most of the evolutionary changes on the sperm occurs within 35 days (Gilber, 1997 ▶). Therefore, all rats in the treatment protocol continued to receive the extract for 30 consecutive days. 

For this purpose, the calculated weight of the extract was selected and then distilled water was added to reach a volume of 1 ml. Then, the animals were administered the extract orally by gavage, daily for 30 consecutive days (Hamza and Amin, 2007 ▶). 

**Morphometric examinations**

One day after the final administration of the extract, the rats were anesthetized by ketamine 10% (80-90 mg/kg) and xylazine 2% (80-90 mg/kg) (Alfasan-Holand) (Afzalzadeh et al., 2013 ▶), then a longitudinal incision was made in the abdomen and scrotum and testis and the attached epididymal ducts were removed with a forceps. To study the testicular texture, the length and width of each testis were measured with a caliper. In order to calculate the volume of the testes, after removing them from the body, testes, cauda epididymis, and vas deferens weight were also measured by means of a digital scale (AND, Japan) with the precision of 0.0001.

**Epididymal sperm count**

According to Afzalzadeh et al. (2013) ▶, after removing the testes and separating the cauda epididymis using a surgical scissor, then placed it into 20 ml of fresh physiological saline at the laboratory temperature, a small incision was created on it to extract the spermatozoa from the tubules. It was homogenized afterwards. After that, 10 ml fresh physiological saline (37 °C) was added to this suspension until an attenuated suspension 10 times (1:200) was produced. One drop of the diluted sperm suspension was transferred to each counting chamber of the improved Neubauer haemocytometer (Deep 1/10 mm, LABART, Munich, Germany) and according to the practical methods for sperm count, white blood cell counts in the regions were recorded and sperms were counted under a light microscope at 100× magnifications.

**Histological studies**

To prepare sections of testicular tissue, several stages were performed, including Bouin fixation of histological specimens, dehydration of samples by increasing concentrations of alcohol, removing the alcohol or clearing up by toluene, paraffin infiltration, molding, and removing the excess paraffin (trimming). Afterward, the paraffin blocks of testicular tissue were prepared using a microtome (SLEE, Germany) and sliced into 5-micron chips. Microscopic sections were stained with hematoxylin and eosin (H&E) and then examined under a light microscope. For germ cell (spermatogonia, spermatocytes, and spermatids) counting as a part of histological evaluation, a camera microscope (Leica, USA) with a 100× magnification. For studying seminiferous tubules diameter, a light microscope (Nikon, Japan) with 40× magnification was used (Vosoughi et al., 2013 ▶).

**Statistical analysis**

Data were analyzed using IBM® SPSS® Data Preparation (version 20.0, New York, USA) software. Data are expressed as mean±standard error of the means (SEM) and were analyzed for statistical significance using one way analysis of variance (ANOVA) followed by LSD test. Results were considered significant at the *p≤*0.05 level.

## Results

**Histological studies**

**Figure 1:**
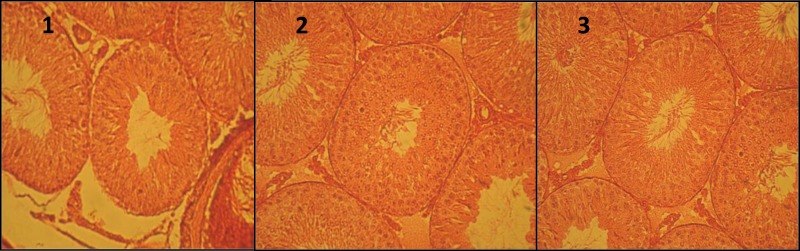
Microscopic slides of different groups: 1 (control), 2 (100 mg/kg of celery extract), and 3 (200 mg/kg of celery extract). Pictures indicate an increase in diameter of seminiferous tubules (DST) and thickness of epithelium in 2 and 3 groups compared with control (H&E,  300).

The results of this study showed that from the statistical point of view, substantial increases were seen in diameter of seminiferous tubules and the spermatocytes, spermatogonia, and spermatozoids (sperms in the cauda epididymis) counts in the treatment groups 2 and 3 in comparisons with the control group (p≤0.05 and p≤0.001, respectively) (Figure 1). 

On the other hand, the number of spermatids was significantly increased in the treatment group 3 (200 mg/kg of celery extract) vs. control group (p≤0.05). However, the spermatid counts showed an increase in the experimental group 2 (100 mg/kg of celery extract) but it was not significant (p>0.05) vs. control group. Moreover, the mean seminiferous tubules diameter (µm) was increased in both experimental groups 2 and 3 but it was only statistically significant in group 3 when compared to control group (p≤0.05) (Table 1).

After 30 days of treatment with aqueous extract of celery, the testis volume was increased significantly in both groups 2 and 3 in comparison with the control group (*p*≤0.001). Moreover, the weights of testes, cauda epididymis and vas deferens were increased but only the weight of epididymis in high dose group (200 mg/kg aqueous extract of celery) was increased statistically significant (*p*≤0.05).

**Table 1:** The effects of aqueous extract of celery on the numbers of spermatids, spermatocytes, spermatogonia, spermatozoids, and the mean of seminiferous tubules diameter

**Groups**	**Spermatis** **(mm** ^2^ **)**	**Spermatocytes** **(mm** ^2^ **)**	**Spermatogonia** **(mm** ^2^ **)**	**Spermatozoids** **(10** ^7^ **)**	**Seminiferous tubules diameter (µm)**
**1 (control)**	13.56±0.05	12.73±0.02	11.57±0.02	3.34±0.04	0.23±0.002
**2 (100mg/kg)**	13.7±0.18	*13.38±0.26	**12.82±0.2	**5±0.17	**0.28±0.002
**3 (200mg/kg)**	*14.2±0.01	*13.44±0.09	**12.78±0.22	**4.96±0.17	**0.28±0.002

(Mean±SEM, n=8).

* p≤0.05,

** p <0.001, compared with control, ANOVA, LSD test.

**Table 2:** The effects of aqueous extract of celery on testis volume and the weights of testis, cauda epididymids, and vas deferens.

**Groups**	**Testis volume** **(mm** ^3^ **10** ^-3^ **)**	**Testis ** **(mg)**	**Cauda epididymis (mg)**	**Vas deferens (mg)**
**1 (control)**	11.58±1.7	1423.6±6.84	409.4±4.64	82±0.89
**2 (100 mg/kg)**	**12.86±1.6	1440±6	412.4±3.66	87.4±5.19
**3 (200 mg/kg)**	**12.92±6.4	1460±4.49	*450.4±5.89	90.8±1.6

(Mean±SEM, n=8).

* p≤0.05,

** p <0.001, compared with control, ANOVA, LSD test.

## Discussion

Medicinal herbs have a great history and credibility. They are considered as precious sources in the history of pharmaceutical science. Moreover, they are known as appropriate alternatives to chemical drugs due to ease of access, fewer side effects, less toxicity, and lower prices (Shamsa et al., 2009 ▶). 

This study suggested that celery extract makes significant changes in the experimental groups when compared with control group, so that oral administration of 100 and 200 mg/kg of extract to male rats for 30 days, caused a significant increase in the diameter of the tubules, the number of spermatogonia, spermatocytes, and spermatozoids as well as testis volume. However, significant increases in the number of spermatids and the weight of cauda epididymis were observed in rats fed by gavage at a dose of 200 mg/kg of celery extract. According to the present study, oral administration of leaf extract of celery can increase the fertility of male rats. These results are compatible with the traditional medicine concepts, indicating that the enhanced sexual performance is achieved by taking the celery extract, in men. Therefore, the increase in testis weight and size in groups received the extract could be attributed to the increase in the number of cells in the testis. Moreover, with the increase of these cells, it could be concluded that these extracts cause an increase in the metabolism of male reproductive tissues. On the other hand, given that the weight of sexual organs can also be affected by sex hormones (Juan et al., 2005 ▶; Khan et al., 2004 ▶), this extract may be effective by affecting the pituitary gland and increasing the sex hormones.

Moreover, given that the process of spermatogenesis and the function of reproductive organs are related to sex hormone secretion, the absorptive and secretory functions of the testes and epididymis could be enhanced (Parandin et al., 2009 ▶; McLachlan et al., 2002 ▶). This may explain the increased number of spermatozoids in the cauda epididymis and the increase in epididymal weight at a high dose of the extract, in addition to increases in size and number of cells in the testes. Since the sperm count is influenced by any change in the absorptive and secretory functions of the testis and epididymis, the increase in this index is considered natural. In a study by Modaresi et al. (2012) ▶ on hydroalchoholic extract of celery leaves, it was shown that injection of celery extract can reduce male reproductive hormones in mice. However, this is contradictory with the traditional use of celery for increasing male sexual performance. This contradiction may be explained by assuming that the effect of the extract is dose-dependent and/or some of toxic compounds may be dissolved better in alcohol than water. Since there are various compounds in an extract, they may change in concentration during passage through the gastrointestinal tract, thus a different impact of celery leaf could be observed on the reproductive system. Another research which was carried out to study the toxic effects of sodium valproate on fertility mentioned that the celery seed extract could effectively reduce the toxicity of sodium valproate in testes tissue. This protective effect of celery seems to be due to its antioxidant property (especially, its apigenin content) and its detoxification capacity (Hamza and Amin, 2007 ▶). Celery effect might be related to the presence of flavonoids, especially apigenin (unpublished data). Apigenin is an antioxidant that is registered as one of the main active compounds in celery (Miean and Mohamed, 2001 ▶). It has also been reported that the cytochrome P450 activity was markedly reduced by celery juice in the liver of Syrian mice (Jakovljevic et al., 2002 ▶).

## Conflict of interest

There is not any conflict of interest in this research. 
